# Anti-oxidant and Anti-Inflammatory Cyclic Diarylheptanoids from *Alnus japonica *Stem Bark

**Published:** 2017

**Authors:** Sabrin R.M. Ibrahim, Gamal A. Mohamed, Amgad I. M. Khedr, Bader M. Aljaeid

**Affiliations:** a *Department of Pharmacognosy and Pharmaceutical Chemistry, College of Pharmacy, Taibah University, Al Madinah Al Munawarah 30078, Saudi Arabia*.; b *Department of Pharmacognosy, Faculty of Pharmacy, Assiut University, Assiut 71526, Egypt. *; c *Department of Natural Products and Alternative Medicine, Faculty of Pharmacy, King Abdulaziz University, Jeddah, 21589, Saudi * *Arabia.*; d *Department of Pharmacognosy, Faculty of Pharmacy, Al-Azhar University, Assiut Branch, Assiut 71524, Egypt*.; e *Department of Pharmacognosy, Faculty of Pharmacy, Port Said University, Port Said 42526, Egypt*.; f *Department of Pharmaceutics and Industrial Pharmacy, Faculty of Pharmacy, King Abdulaziz University, Jeddah 21589, Saudi Arabia*

**Keywords:** *Alnus japonica*, Alnuheptanoid B, Cyclic diarylheptanoid, Antioxidant, Anti-inflammatory

## Abstract

A new cyclic diarylheptanoid namely alnuheptanoid B (3), along with four known cyclic diarylheptanoids: myricanone (1), (+)-*S*-myricanol (2), myricanone 5-*O*- -D-glucopyranoside (4), and (+)-*S*-myricanol 5-*O*- -D-glucopyranoside (5) were isolated from the EtOAc fraction of *Alnus japonica* Steud (family: Betulaceae) stem bark. Their structures were established by different spectroscopic analyses, as well as optical rotation measurement. Compounds 1, 2, 4, and 5 are isolated for the first time from *A*.* japonica*. The antioxidant and anti-inflammatory activities of compounds (1-5) were assessed using DPPH assay and carrageenin induced rat paw edema model, respectively. They displayed significant antioxidant activity in relation to propyl gallate (standard antioxidant) at concentration 50 *µ*M. Compound 2 demonstrated anti-inflammatory effect at a dose 10 mg/kg compared with indomethacin (positive control).

## Introduction

**Figure 1:**
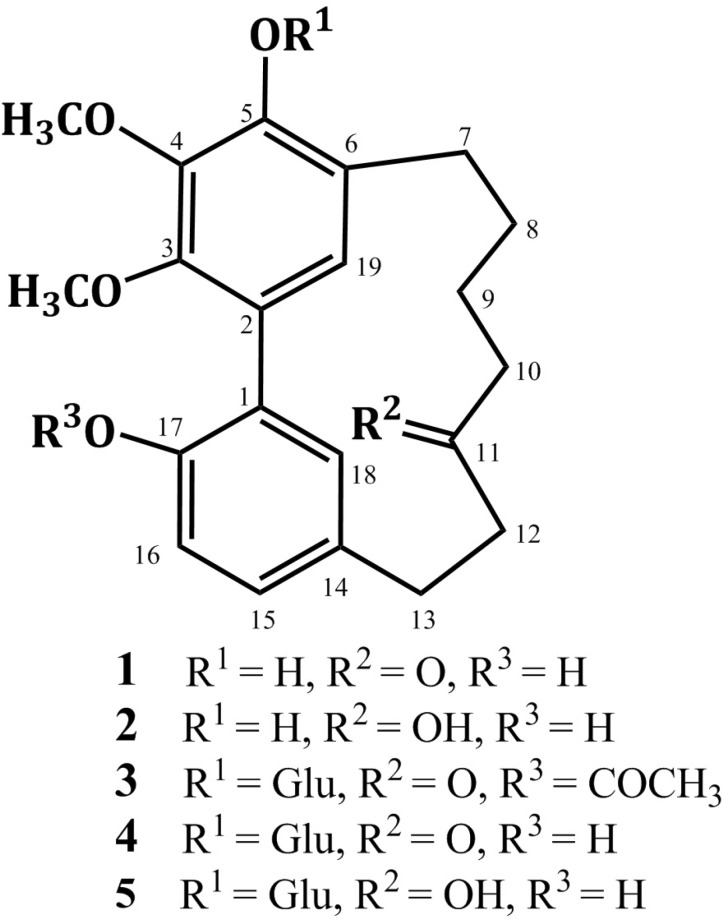
Structures of compounds **1**-**5**.

Diarylheptanoids have been isolated from various genera such as *Acer* (Aceraceae), *Platycarya *(Juglandaceae), *Myrica *(Myricaceae), *Centrolobium* (Leguminosae), *Alpinia*, *Curcuma*, and *Zingiber* (Zingiberaceae), and *Alnus* and *Betula *(Betulaceae) (1, 2). The structure of diarylheptanoids consists of two benzene rings linked by a linear C_7_-aliphatic chain with varying functional groups on the aryl and aliphatic moieties. They can be sub-grouped into open chain linear or cyclic compounds (2, 3). The latter group includes *meta*-*meta* bridged biphenyls and *meta*-*para* diphenyl ethers (2, 3). In addition, more complex diarylheptanoids with the basic skeleton extended by fragments such as arylbutyl, chalcone or flavonoid moieties have been isolated (4). They showed wide variety of biological activities as antioxidant, anti-inflammatory, antitumor, neuro-protective, estrogenic, hepatoprotective, anti-influenza, anti-trypanosomal, antiviral, and leishmanicidal (3, 5-15). In Japan, *Alnus japonica* (Betulaceae) is widely distributed in the low mountains (12, 13). Previous phytochemical study of *A*.* japonica *revealed the presence of diarylheptanoids, triterpenoids, flavonoids, and hydrolysable tannins (12-22). Continuing our study on *A*.* japonica *stem bark resulted in the isolation and characterization of alnuheptanoid B, a new cyclic diarylheptanoid, along with four known cyclic diarylheptanoids (Figure 1, Tables 1 and 2). They were assessed for their free radical scavenging activity using DPPH assay. Also, their anti-inflammatory effect was estimated using carrageenin induced paw edema method.

## Experimental

*General*

Melting points were determined by Electrothermal Digital Melting Point 9100 instrument (England). Shimadzu 1601 UV/VIS and Shimadzu Infrared-400 (Japan) spectrophotometers were used to measure the UV and IR spectra, respectively. Optical rotation was measured by Perkin-Elmer Polarimeter 341 LC Model (USA). LRMS spectra were assessed by a MATTSQ7000 Finnigan spectrometer. A Micromass Qtof 2 spectrometer was used for HRESIMS spectra measurements. Bruker Avance DRX 400 (Bruker, USA) was used to record NMR spectra. HPLC separations were performed on a HPLC system consisting of a UV L-7400 detector (280 nm) and a Lachrom-Merck L-7100 Hitachi pump using a C_18_ column (250 × 8 mm i.d., Eurospher 100, Germany). A linear gradient (H_2_O:MeOH 80:20 % to MeOH 100 % over 45 min) was applied. Chromatographic separation was achieved using RP_18_ (0.04-0.063 mm) and silica gel (0.04-0.063 mm) 60 (Merck, Germany). The TLC analysis was performed using the following systems: MeOH:CHCl_3_ (10:90, S_1_), MeOH:CHCl_3_ (15:85, S_2_), and *n*-BuOH:H_2_O:HOAc (4:5:1, S_3_). Pre-coated silica gel 60 F_254_ TLC plates (0.2 mm, Merck) was used for TLC. Propyl gallate (PG), 2,2-diphenyl-1-picrylhydrazyl (DPPH), carrageenin, and indomethacin were provided by Sigma-Aldrich Co. (Taufkirchen, Germany). 

*Plant material*

The plant sample was obtained from the Heinrich-Heine University`s botanical garden (Düsseldorf, Germany) in March 2005. The plant was taxonomically authenticated and identified by Peter Westhoff, Prof. of Plants Molecular Biology and Development (Heinrich-Heine University, Germany). A specimen (Registration code AJB-2005) was kept at the Faculty of Pharmacy, Department of Pharmacognosy, Al-Azhar University, Egypt. 

*Extraction and isolation*

The powdered stem bark (200 g) was extracted exhaustively with EtOH (70 %, 4 × 2 L). The EtOH extract was concentrated to afford 12 g brown residue. The residue was chromatographed over vacuum liquid chromatography (VLC) using 50 % CHCl_3_:*n*-hexane (500 mL x 4) and EtOAc (500 mL x 4) to obtain 2.6 and 4.1 g, respectively. The VLC of the EtOAc (4.1 g) fraction using CHCl_3_:MeOH gradient elution afforded 6 sub-fractions (A-F). Sub-fractions B-E were previously investigated by authors (13). SiO_2_ column (2 cm 50 cm 50 g) of sub-fraction A (470 mg, CHCl_3_:MeOH 90:10 v/v) with CHCl_3_:MeOH gradient, followed by semi-preparative HPLC yielded 1 (22.1 mg, white needles) and 2 (14.8 mg, white needles). RP-18 column (2 cm 50 cm 60 g) of sub-fraction F (392 mg, CHCl_3_:MeOH 40:60 v/v) using MeOH:H_2_O gradient elution followed by HPLC gave 3 (13.7 mg), 4 (15.3 mg), and 5 (18.9 mg). 

*Myricanone* *(1)*

White needles (22.1 mg); m.p. 191-192 °C; UV (MeOH) _max_: 215, 258, 295 nm; NMRdata: see Table 1; ESIMS: *m*/*z* 357 [M + H]^+^.

*S-* *Myricanol* *(2)*

White needles (14.8 mg); m.p. 103-104 °C; [α]_D_ +38.5 (*c *0. 5, CHCl_3_); UV (MeOH) _max_: 221, 259, 295 nm; NMR data: see Table 1; ESIMS: *m*/*z* 359 [M + H]^+^.

*Alnuheptanoids* *B (3)*

White amorphous powder (13.7 mg); UV (MeOH) _max_ (log  ): 219 (4.71), 251 (4.36), 293 (3.95) nm; IR (KBr)  _max_: 3465, 1725, 1715, 1598 cm^-1^; NMR data: see Table 2; ESIMS: *m*/*z* 561 [M + H]^+^, 398.9 [(M + H)-Glu]^+^, 357.2 [(M + H)-(Glu+Acetyl)]^+^; HRESIMS: *m*/*z* 561.2339 (calc for C_29_H_37_O_11_, 561.2336 [M + H]^+^).

*Myricanone* *5-O--D-glucopyranoside (4)*

White amorphous powder (15.3 mg); UV (MeOH) _max_: 220, 251, 294 nm; NMR data: see Table 2; ESIMS: *m*/*z* 519 [M + H]^+^, 357 [(M + H)-Glu]^+^.

*(+)-S-* *Myricanol* *5-O--D-glucopyranoside (5)*

 White amorphous powder (18.9 mg); [α]_D_ +82.3 (*c *0. 5, CH_3_OH); UV (MeOH) _max_: 231, 253, 294 nm; NMR data: see Table 2; ESIMS: *m*/*z* 521 [M + H]^+^, 359 [(M + H)-Glu]^+^.

*Antioxidant activity*

The antioxidant effect of compounds 1-5 was evaluated by 2,2`-diphenylpicrylhydrazyl (DPPH) assay as previously outlined (23, 24). 

*Carrageenin-induced rat paw edema method*

The anti-inflammatory activity was evaluated on adult male albino rats (120-150 g b. wt.) using the same procedures as previously described (23, 25 ,26).

*Statistical analysis*

All data were expressed as mean ± standard error of mean using the student *t* test. ANOVA (one-way analysis of variance) was used for evaluation of statistical significance. The values were considered to be significantly different when *P* < 0.01.

## Results and Discussion

**Figure 2:**
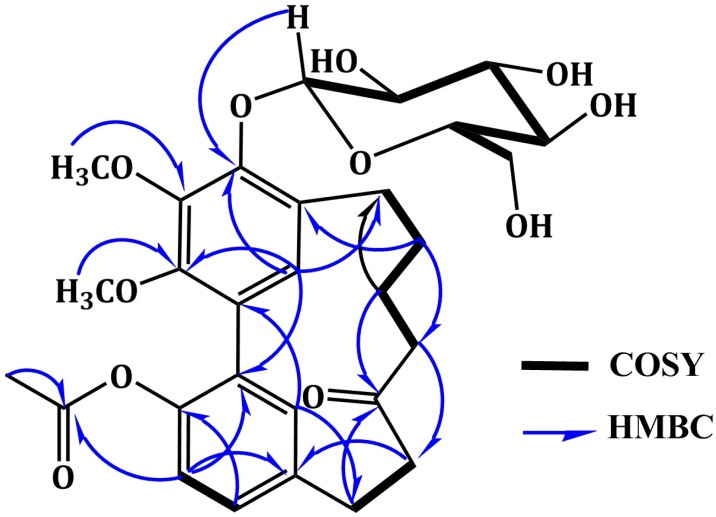
Important ^1^H-^1^H COSY and HMBC correlations of alnuheptanoid B (**3**).

Compound 3 was isolated as white amorphous powder. A molecular formula C_29_H_36_O_11 _was established from the HRESIMS quasi-molecular ion peak at *m*/*z* 561.2339 [M + H]^+^. The IR, UV, and NMR spectral data of 3 were in agreement with those of 4 except for the appearance of new signals at _H_ 2.08 /_C_ 20.8 (COCH_3_) and 171.7 (COCH_3_) characteristic for an acetyl group in 3. Its attachment at C-17 was confirmed by the ^3^*J* HMBC cross peak of H-16 to the carbonyl group of acetyl moiety at _C_ 171.7 and further secured by the ESIMS ion peak at 357.2 [(M + H)-(Glu+Acetyl)]^+^. In addition, 3 was 42 mass units and one degree of unsaturation more than 4, confirming the presence of the acetyl moiety. The UV absorption maxima at 219, 251, and 293 nm indicated a diarylheptanoid structure of 3 (27). Its IR spectrum displayed bands ascribable to hydroxyl (3465 cm^-1^), ester carbonyl (1725 cm^-1^), ketone carbonyl (1715 cm^-1^), and benzene (1598 cm^-1^) functionalities (28). The ^1^H NMR spectrum showed two singlet methoxy groups at _H_ 3.95 (4-OCH_3_) and 3.79 (3-OCH_3_) (Table 2). They correlated with the carbons resonating at _C _61.9 (4-OCH_3_) and 61.7 (3-OCH_3_), respectively in HMQC spectrum. Their connectivity at C-4 and C-3 was proven by the HMBC cross peaks of 4-OCH_3_ to C-4 (_C_ 145.0) and 3-OCH_3_ to C-3 (_C_ 146.9). Four aromatic proton signals at _H_ 7.06 (dd, *J* = 6.6, 1.5 Hz, H-15), 6.87 (d, *J* = 6.6 Hz, H-16), 6.69 (d, *J* = 1.5 Hz, H-18), and 6.67 (s, H-19), which correlated with carbons at _C_ 129.6 (C-15), 122.8 (C-16), 132.4 (C-18), and 129.1 (C-19) in HMQC spectrum, indicating the presence of 1,2,4-*tri*-substituted and 1,2,3,4,5-*penta*-substituted benzene moieties in 3 (20,21). They were established by the COSY cross peaks of H-15 to H-16 and H-18 and further secured by the HMBC correlations of H-15 to C-17 and C-18, H-16 to C-1 and C-14, H-18 to C-14 and C-17, and H-19 to C-3 and C-5 (Figure 2). The connectivity of two phenyl moieties at C_1_-C_2_ was secured based on the HMBC cross peaks of H-19 to C-1 and H-18 to C-2 (Figure 2). Moreover, the doublet proton signal at _H_ 4.80 (*J* = 7.6 Hz, H-1`) showed cross peak to the signal at _C_ 105.0 (C-1`), indicating the presence of *β*-glucopyranose moiety (23, 26). This was established by the observed ESIMS fragment peaks at *m*/*z* 398.9 [(M + H)-Glu]^+^ and 357.2 [(M + H)-(Glu+Acetyl)]^+^. In the HMBC, the cross peak of H-1` to C-5 (_C_ 148.7) established the placement of glucose at C-5. Furthermore, signals for six methylene groups at _H_ 1.80-3.07 and ketone carbonyl at _C_ 213.6 (C-11), characteristic for heptanoid moiety in 3 were observed. In the COSY spectrum, the spin system started from H-7 to H-10 and cross peak of H-12 to H-13 established this moiety. It was secured by the observed HMBC cross peaks of H-9/C-7 and C-11, H-10/C-8, H-12/C-13, and H-13/C-11. The attachment of heptanoid moiety at C6-C14 of the biphenyl moiety was secured by the HMBC cross peaks of H-8/C-6, H-19/C-7, and H-18/C-13. Consequently, 3 was concluded to be 17-*O*-acetyl myricanone 5-*O*-*β*-glucopyranoside and named alnuheptanoid B.

Compounds 1-5 were identified to be myricanone (1) (29, 30), (+)-*S*-myricanol (2) (30,31), myricanone 5-*O*- -D-glucopyranoside (4) (32), and (+)-*S*-myricanol 5-*O*- -D-glucopyranoside (5) (27) by the interpretation of the spectroscopic data and comparison with literature.

**Table 1:** ^1^H and ^13^C NMR data of compounds 1 and 2 (400 and 100 MHz, CDCl_3_).

**No.**	**1**		**2**	
	_H _[m, *J* (Hz)]	_C _(Mult.)	_H _[Mult., *J* (Hz)]	_C _(Mult.)
1	-	125.4 C	-	124.7 C
2	-	123.1 C	-	122.6 C
3	-	145.9 C	-	145.8 C
4	-	138.7 C	-	138.6 C
5	-	147.8 C	-	147.7 C
6	-	123.0 C	-	123.4 C
7	2.72 m	26.8 CH_2_	2.55 m 1.93 m	25.7 CH_2_
8	1.94 m	24.4 CH_2_	2.78 m 1.92 m	25.4 CH_2_
9	1.87 m	21.8 CH_2_	1.69 m 1.55 m	23.0 CH_2_
10	2.77 m	46.1 CH_2_	1.90 m 1.54 m	39.4 CH_2_
11	-	213.6 C	4.08 m	68.6 CH
12	2.81 m	42.5 CH_2_	2.33 m 1.72 m	34.7 CH_2_
13	3.03 m	28.8 CH_2_	2.94 m	26.9 CH_2_
14	-	132.4 C	-	130.6 C
15	7.05 dd (6.6, 2.0)	128.9 CH	7.08 dd (7.0, 1.5)	129.9 CH
16	6.88 d (6.6)	116.9 CH	6.91 d (7.0)	116.8 CH
17	-	151.7 C	-	151.4 C
18	6.74 d (2.0)	132.4 CH	7.17 d (1.5)	133.1 CH
19	6.60 s	128.9 CH	6.90 s	129.4 CH
3-OCH_3_	3.81 s	61.3 CH_3_	3.87 s	61.3 CH_3_
4-OCH_3_	3.98 s	61.4 CH_3_	3.99 s	61.4 CH_3_
17-OH	7.66 brs	-	7.70 brs	-
OH	5.91 brs	-	5.90 brs	-

**Table 2:** ^1^H and ^13^C NMR data of compounds 3-5 (400 and 100 MHz, DMSO-*d*_6_).

**No.**	**3**		**4**		**5**	
	_H _[m, *J* (Hz)]	_C _(Mult.)	_H _[Mult., *J* (Hz)]	_C _(Mult.)	_H _[Mult., *J* (Hz)]	_C _(Mult.)
1	-	130.9 C	-	128.9 C	-	128.4 C
2	-	128.9 C	-	128.0 C	-	128.0 C
3	-	146.9 C	-	148.5 C	-	148.3 C
4	-	145.0 C	-	145.3 C	-	145.1 C
5	-	148.7 C	-	148.7 C	-	148.8 C
6	-	124.8 C	-	126.1 C	-	126.0 C
7	2.82 m 2.72 m	27.8 CH_2_	2.81 m	27.1 CH_2_	2.54 m	25.8 CH_2_
8	1.90 m 1.86 m	24.7 CH_2_	1.75 m	24.2 CH_2_	2.71 m	26.0 CH_2_
9	1.80 m	21.9 CH_2_	1.51 m	21.2 CH_2_	1.28 m 1.21 m	22.5 CH_2_
10	2.75 m 2.67 m	45.8 CH_2_	2.63 m	45.1 CH_2_	1.63 m 1.35 m	39.3 CH_2_
11	-	213.6 C	-	213.2 C	3.95 m	66.5 CH
12	2.77 m	42.4 CH_2_	2.74 m	41.7 CH_2_	2.09 m 1.49 m	34.4 CH_2_
13	3.07 m 2.95 m	28.6 CH_2_	2.84 m	28.0 CH_2_	2.83 m 2.78 m	26.8 CH_2_
14	-	132.1 C	-	130.7 C	-	129.4 C
15	7.06 dd (6.6, 1.5)	129.6 CH	6.95 dd (6.6, 1.7)	128.3 CH	6.96 dd (6.6, 1.5)	129.1 CH
16	6.87 d (6.6)	122.7 CH	6.71 d (6.6)	115.5 CH	6.74 d (6.6)	115.6 CH
17	-	149.8 C	-	152.3 C	-	152.0 C
18	6.69 d (1.5)	132.4 CH	6.45 d (1.7)	133.2 CH	6.92 brs	134.6 CH
19	6.67 s	129.1 CH	6.35 s	128.5 CH	6.60 s	129.5 CH
1`	4.80 d (7.6)	105.0 CH	4.79 d (7.6)	103.4 CH	4.84 d (7.6)	103.9 CH
2`	3.33 m	74.1 CH	3.19 m	74.0 CH	3.04 m	74.0 CH
3`	3.37 m	77.2 CH	3.06 m	77.0 CH	3.06 m	77.1 CH
4`	3.95 m	69.9 CH	3.16 m	69.9 CH	3.17 m	69.9 CH
5`	3.67 m	76.3 CH	3.24 m	76.4 CH	3.23 m	76.5 CH
6`	3.86 m 3.70 m	62.0 CH_2_	3.61 m 3.43 m	61.0 CH_2_	3.58 m 3.42 m	60.9 CH_2_
3-OCH_3_	3.79 s	61.7 CH_3_	3.75 s	60.1 CH_3_	3.81 s	60.1 CH_3_
4-OCH_3_	3.95 s	61.9 CH_3_	3.81 s	60.9 CH_3_	3.83 s	60.8 CH_3_
17-OH	-	-	8.91 brs	-	-	-
2`-OH	-	-	5.03 brs	-	5.03 d (2.5)	-
3`-OH	-	-	4.93 d (4.3)	-	4.93 d (4.1)	-
4`-OH	-	-	4.09 d (4.3)	-	4.41 d (3.8)	-
5`-OH	-	-	5.28 d (3.5)	-	5.22 d (3.5)	-
6`-OH	-	-	4.34 t (4.6)	-	3.36 t (4.6)	-
17-COCH_3_	2.08 s	20.8 CH_3_ 171.7 C	-	-	-	-

The antioxidant activity of the isolated cyclic diarylheptanoids (1-5) was determined using DPPH free radical scavenging system at concentration 50 *µ*M. The results showed that, 1 and 2 had significant antioxidant activity. While, 3-5 showed moderate activity in comparison with propyl gallate (a known antioxidant) (Table 3). Their antioxidant effect was related to the number of free hydroxyl groups in their structures. Compounds 1 and 2 showed significant activities compared to propyl gallate at the same concentration. However, blocking of the hydroxyl group by an acetyl or glucose moiety leads to a decrease in the activity as in 3-5 (33).

**Table 3:** The DPPH radical scavenging activity results.

**Sample**	**DPPH (% Inhibition)**
1	63.10 ± 0.81
2	70.14 ± 0.55
3	41.16 ± 0.64
4	49.09 ± 0.76
5	52.11 ± 0.59
Propyl gallate	97.31 ± 0.37

Conc. 50 µM; Each value represents the mean ± S.D.; *n* = 3.

Compounds 1-5 were evaluated for their anti-inflammatory effects using carrageenin induced paw edema model. Compound 2 showed the highest activity comparable to indomethacin (10 mg/kg) (Table 4). Also, 1, 3, 4, and 5 showed potent activity at dose 10 mg/kg after 4 h. The phenolic compounds are known to inhibit prostaglandins synthesis enzymes, more specifically the endoperoxide (26). It was reported that, prostaglandin like substances are released during the second phase of carrageenin induced edema (34, 35). So, the anti-inflammatory effects of the tested compounds may be due to inhibition of prostaglandin like substances.

**Table 4:** The anti-inflammatory activity results.

Groups n = 6	Dose mg/kg		0 hr		1 hr		2 hr		4 hr		6 hr	
			PET^a^	% IN^b^	PET^a^	% IN^b^	PET^a^	% IN^b^	PET^a^	% IN^b^	PET^a^	% IN^b^
Inflamed			3.95±0.09	0.00	5.76±0.12	0.00	6.51±0.11	0.00	6.97±0.10	0.00	4.88±0.13	0.00
Inflamed + Indom.c		10	3.94±0.14	0.25	5.39±0.14*	6.42	3.41±0.15*	47.62	3.12±0.08*	55.24	2.99±0.07*	38.73
Inflamed + **1**		10	3.89±0.12	1.52	3.97±0.11*	29.98	3.77±0.12*	42.09	3.51±0.07*	49.64	3.24±0.11*	33.61
Inflamed + **2**		10	3.87±0.07	2.03	3.94±0.10*	31.60	3.62±0.06*	44.39	3.38±0.13*	51.51	3.08±0.12*	36.89
Inflamed + **3**		10	3.93±0.12	0.51	4.37±0.10*	24.13	3.99±0.13*	38.71	3.74±0.11*	46.34	3.49±0.13*	28.48
Inflamed + **4**		10	3.94±0.14	0.25	4.32±0.15*	25.00	3.91±0.11*	39.94	3.70±0.09*	46.92	3.42±0.15*	29.92
Inflamed + **5**		10	3.91±0.08	1.01	4.07±0.14*	29.34	3.87±0.09*	40.55	3.69±0.11*	47.06	3.31±0.08*	32.17

Each value represents the mean ± S.E.M., *n* = 6;

* Significant different from inflamed control group at *P* < 0.01;

a PET: Paw edema thickness;

b % IN: % Inhibition;

c Indom: Indomethacin.

The observed activity of these compounds might be through the inhibition of the inflammatory prostanoids (36, 37). In this work, we can make a conclusion on the SAR of the tested cyclic diarylheptanoids. It was observed that, the phenolic hydroxyl groups are responsible for anti-inflammatory activity as in 1 and 2 (36, 37). Glucosidation of phenolic OH group leads to reduce the activity as in 3-5. Secondary alcoholic hydroxyl group in aliphatic chain might increase the activity as in 1 and 5 in comparison to the other compounds. 

## Conclusions

A new cyclic diarylheptanoid and four known compounds were isolated from *A*.* japonica* for the first time. Their chemical structures were established by different spectroscopic analyses. Compounds 1 and 2 showed significant antioxidant activity. Compound 2 exhibited potent anti-inflammatory activity.
